# Artificial Intelligence in Wearable ECG and PPG Devices for Arrhythmia Detection: A Narrative Review: Short title: Artificial Intelligence in Wearable Devices for Arrhythmia Detection

**DOI:** 10.31661/gmj.v15i.4097

**Published:** 2026-09-18

**Authors:** Farid Taghavi, Shirin Alord, Alireza Ghaffari, Somayye Mehanfar, Kamran Mohammadi

**Affiliations:** ^1^ Cardiovascular Research Center, Tabriz University of Medical Sciences, Tabriz, Iran; ^2^ Cardiovascular Research Center, Health Policy and Promotion Institute, Kermanshah University of Medical Sciences, Kermanshah, Iran; ^3^ Faculty of Electrical and Computer Engineering, University of Tabriz, Tabriz, Iran; ^4^ Student Research Committee, Tabriz University of Medical Sciences, Tabriz, Iran

**Keywords:** Artificial Intelligence, Wearable Devices, Arrhythmia Detection, ECG, PPG

## Abstract

Cardiovascular disease is the world’s top cause of death. Short recording times and a dependence on clinical settings are two practical drawbacks of traditional methods like Holter monitoring and standard 12-lead ECG. Two significant trends have come together in recent years: the broad availability of consumer-grade wearable technology, including smartwatches and adhesive ambulatory ECG patches, and the quick advancement of deep learning and artificial intelligence. Four main themes currently dominate research in this field: diagnostic accuracy under controlled conditions, algorithmic innovation for real-time and on-device analysis, real-world implementation and performance in large prospective studies, and the persistent technical, clinical, and regulatory challenges that limit widespread deployment.AI-based systems have shown consistently high performance in controlled environments and curated datasets, with reported sensitivities and specificities commonly ranging between 92% and 99%.However, performance, especially specificity and generalizability, frequently declines significantly if these algorithms are exposed to the messiness of daily movement, a variety of patient demographics, fluctuating skin contact, and hardware limitations outside of the lab. More advanced transformer architectures and multimodal techniques that combine ECG signals with photoplethysmography (PPG) waveforms, as well as lightweight convolutional neural networks that can operate directly on the device itself and provide true real-time detection, have been the focus of recent algorithmic work. The present status of wearable ECG and PPG technology for AI-enabled arrhythmia detection is examined in this narrative review. It critically evaluates diagnostic performance, translational gaps that still exist and paying special attention to the major prospective studies that have shaped the field. All things considered, the data indicates that wearables driven by AI have real potential for screening for arrhythmias at the population level and have already shown promising signs of clinical utility. The field will require larger, longer, and more diversified prospective trials that are sufficiently powered to identify significant differences in hard clinical outcomes.

## Introduction

Cardiovascular diseases (CVDs) is the leading cause of death worldwide, as evidenced by the increase in deaths from CVDs from over 12.4 million in 1990 to nearly 19.8 million in 2022. Because of this steadily increasing burden, which is mostly caused by demographic growth, ageing populations, and modifiable risk factors, it is imperative that early, precise, and broadly applicable diagnostic techniques be developed [1]. Standard 12-lead ECGs and 24-hour Holter recorders are two examples of conventional cardiac rhythm monitoring instruments that are still limited by their brief capture windows and reliance on supervised clinical settings. Therefore, silent or paroxysmal arrhythmias often remain undetected during the short observation periods [2, 3]. The gap between sporadic snapshots and actual rhythm abnormalities is now significantly reduced due to artificial intelligence’s ability to continuously and automatically sort through massive streams of ECG data[4].

Continuous, real-time rhythm surveillance has become a part of daily life as a result of the most recent generation of wearable ECG devices, which range from consumer smartwatches to adhesive chest patches. These devices have reported significantly higher detection rates for atrial fibrillation and other clinically significant arrhythmias when they integrate AI directly into ECG sensing, which is still the unquestioned gold standard for arrhythmia diagnosis[5, 6]. Currently, the previous model of episodic, clinic-based testing is gradually being replaced by seamless, population-level cardiac screening. However, significant obstacles still persist. Signal quality can be substantially degraded by patient movement, poor electrode / skin interface, and inter-individual physiological variability[7]. Furthermore, biases in the datasets that many algorithms are trained on continue to exist [8]. Technical difficulties, a lack of complete clinical trust, regulatory obstacles, and insufficient integration into current care pathways are some of the problems that continue to prevent widespread clinical application[9].

Evidence in this field has now moved from small feasibility studies and retrospective analyses to Large-scale real-world data gathering and analytic studies with over 1.5 million participants that have been collected through several major prospective studies; including the Apple Heart Study (n = 419,297), the Fitbit Heart Study with its 2024 long-term follow-up, the Huawei Heart Study, the Lux-Dx INSERT trial, the 2024 Cardiologs AI validation study on single-lead Apple Watch ECGs[10] , and STROKESTOP II [5]. These studies have become the most reliable data currently available from unselected ambulatory populations on diagnostic yield, patient adherence, positive predictive value, and downstream clinical outcomes [3].

Consequently, four primary Research Questions (RQs) serve as the framework for the current narrative review:

• When tested on carefully selected and openly accessible ECG and PPG datasets, how accurate are AI-based algorithms for identifying atrial fibrillation and other clinically significant arrhythmias?

• Which recent algorithmic developments, given the computational and energy limitations of wearable devices, allow for the detection of arrhythmias in real time or on-device? [6, 11].

• What can be learnt about the practical application, user compliance, and clinical efficacy of wearable arrhythmia detection powered by AI from extensive prospective population-based studies? [5, 12].

• What are the main clinical, technical, and legal barriers that still prevent these technologies from being routinely deployed and translated into large-scale clinical settings? [13, 14].

Our objective is to synthesise the full range of evidence, from highly controlled database studies to large-scale real-world deployments, and explain how the literature was found and selected in order to gather and present the beneficial implications for future research and clinical practice [15].

## Method

### Design and reporting

Focusing primarily on atrial fibrillation and other clinically significant arrhythmias, this narrative review thematically assesses and synthesizes the available evidence using a predefined search strategy and selection protocol. It examines the application of artificial intelligence and deep learning techniques to single-lead ECG and photoplethysmography (PPG) signals obtained from both consumer-grade and medical-grade ambulatory wearable devices. The review follows the SANRA guidelines for high-quality narrative reviews and transparently reports all methodological steps [16].

### Timeframe and data sources

The following databases were searched between January 2015 and August 2025: PubMed/MEDLINE, Scopus, Core Collection of the Web of Science, IEEE Xplore[SR1] In addition, Google Scholar was utilized to track forward and backward citations of important papers and to retrieve difficult-to-access full texts [17].

### Search methodology

Artificial intelligence approaches (artificial intelligence, AI, “machine learning,” “deep learning,” “neural network,” etc.); wearable platforms (smartwatch, “wearable ECG,” “ECG patch,” “wrist-worn,” “fitness tracker,” etc.); and cardiac endpoints (electrocardiogram, ECG, photoplethysmography, PPG, “atrial fibrillation,” AFib, “arrhythmia detection,” sensitivity, specificity, AUC, etc.) were combined using a Boolean search. On request, the authors will provide the full search strings. 456 records were found during the initial database search [5].

### Eligibility criteria

Original studies were included if they met the following criteria: (a) they applied artificial intelligence, machine learning, or deep learning algorithms to single-lead ECG or PPG signals acquired from wrist-worn smartwatches, adhesive chest patches, and other ambulatory wearable devices in adult populations undergoing wearable-based cardiac monitoring [4]; (b) they reported diagnostic performance measures including sensitivity, specificity, AUC/ROC, accuracy, F1-score, and positive predictive value for atrial fibrillation or other clinically relevant arrhythmias, typically assessed in comparison with conventional clinical ECG-based monitoring [6]; and (c) they used a clinically validated reference standard including 12-lead ECG, Holter monitoring, medical-grade ambulatory patches, and implantable loop recorders to evaluate diagnostic efficacy and real-world applicability [3]. Studies based solely on signal simulation, non-arrhythmic endpoints, stationary monitoring systems, or isolated signal reconstruction were excluded.

### Study selection and data extraction

Following duplicate removal, the titles and abstracts of 408 unique records were screened. Full-text review was then performed for all potentially eligible studies. In addition to the database search, several large-scale prospective real-world studies were identified through manual reference checking to ensure comprehensive coverage of high-level evidence. A small number of records were excluded at this stage due to non-arrhythmic endpoints or the absence of arrhythmia-specific performance data. Ultimately, 20 studies were selected for inclusion in the final narrative synthesis [12]. Study selection and data extraction were performed independently by two reviewers, with discrepancies resolved by consensus and rechecking. Key information, including study design, participant characteristics, device type, signal modality, reference standard, diagnostic performance measures, adherence outcomes, and major limitations, was recorded in structured summary tables.

### Synthesis

Based on our main four RQs, four according thematic categories were used to arrange the evidence for narrative synthesis: diagnostic accuracy attained on publicly available and curated datasets [15]; algorithmic advancements that allow for on-device or real-time inference [18]; implementation and performance results from the historic perspective population-scale trials [5]; and obstacles and translational difficulties faced during practical ambulatory use [14].

## Findings

Twenty pertinent studies that were published between 2019 and 2025 provided the evidence. The Apple Heart Study [2], the Fitbit Heart Study with long-term follow-up reports [5], the Huawei Heart Study [19], the Lux-Dx INSERT trial [12], the Cardiologs AI single-lead ECG validation using Apple Watch Series 4–8 devices [10], and STROKESTOP II [20] were all manually identified and included to ensure the highest level of clinical evidence [2, 5, 10, 12, 19, 20].

Systematic database searches in PubMed, Embase, Scopus, and Web of Science were used to find all of the remaining studies. Titles, abstracts, and full-text records were then systematically screened. Studies that used artificial intelligence or deep learning techniques to detect or predict atrial fibrillation or other clinically significant arrhythmias using single-lead ECG or photoplethysmography (PPG) signals obtained from consumer wearable devices were the only ones that qualified [19].

Four thematic categories were used to group the final set of twenty studies: (A) groundbreaking large-scale prospective consumer PPG/ECG trials and outcome studies [2, 5]; (B) lightweight algorithmic innovations optimised for on-device or edge deployment [8]; (C) foundation-scale and multimodal pre-trained models directly relevant to future wearable applications [21]; and (D) small- to medium-sized real-world deployments, prototype validations, and predictive modelling studies [7].

Four main themes are addressed by these evidence streams taken together: algorithmic advancements under wearable device computational constraints, real-world performance in large prospective trials [3, 5], diagnostic performance in controlled datasets [9], and the remaining clinical, technical, and regulatory obstacles to large-scale clinical translation [13, 14]. Because of the significant heterogeneity across device types, populations, signal modalities, and reported outcomes, narrative synthesis was chosen over quantitative meta-analysis.

1. Landmark Large-Scale Prospective Consumer Trials and Outcome Studies

 Theme 1 findings represents large-scale prospective studies that have evaluated atrial fibrillation (AF) detection using consumer-grade wearables, single-lead ECG devices, and implantable cardiac monitors in real-world settings. These studies reflect the field’s progressive shift from controlled technical algorithm validation toward population-scale screening and outcome-oriented detection strategies [2, 4, 5]. The increased use of wearables based on photoplethysmography (PPG), single-lead ECG timepieces, and continuous implanted cardiac monitoring systems has made it possible for AF surveillance to move from clinical settings into daily life [2, 5, 7]. In order to offer an organized framework for cross-study comparison, Table1 incorporates important factors such as population size, signal modality, AF confirmation approach, diagnostic performance, and practical constraints. The results of theme 1 studies characteristics are presented in Table1.

Table1 shows how AF detection strategies have evolved across a broad technological and clinical spectrum, from mass consumer screening with PPG-based wearables to continuous implantable ECG monitoring and outcome-driven ECG screening programs. At the population scale, the Apple Heart Study, Huawei Heart Study, and Fitbit Heart Study collectively demonstrate that PPG-based screening is feasible in cohorts exceeding 180,000 to over 450,000 individuals [2, 4, 5]. These studies differ in how AF is defined and confirmed. The Apple Heart Study combined irregular pulse notification with subsequent ECG patch confirmation and achieved extremely high sensitivity (98.3%) and specificity (99.6%) [2], yet only 0.5% of users ultimately received confirmed AF, highlighting the tension between large-scale reach and low event yield. Fitbit adopted a more conservative IHRD definition requiring at least 30 minutes of sustained irregularity prior to ECG confirmation, which produced an equally high PPV (98%) and specificity (99.7%) [5] but at the cost of missing shorter AF episodes. In contrast, the Huawei Heart Study emphasized feasibility in the general population and reported a high PPV (91.6%), although the lack of sensitivity and specificity metrics limits direct performance comparison with the Apple and Fitbit cohorts [4].

Earlier clinical validation is represented by the WATCH AF Trial, which tested PPG-based smartwatch detection against cardiologist-interpreted iECG in an elderly hospitalized cohort. Despite motion artifacts and a substantial uninterpretable rate due to poor skin contact, sensitivity (93.7%), PPV (97.8%), and specificity (98.2%) remained high, showing that diagnostic performance is preserved even in less controlled clinical environments. This contrasts with later consumer mega-studies that benefited from healthier, younger populations but faced confirmation bottlenecks and lower absolute AF yield.

Beyond wearables, continuous monitoring is addressed by the LUX-Dx PERFORM study, where arrhythmia detection is performed using a subcutaneous implantable cardiac monitor with remote transmission and programming. Although diagnostic accuracy for AF was not yet reported at the interim stage, the study demonstrated a low infection rate and high daily data transmission success, emphasizing feasibility and safety rather than population-level screening efficiency [12]. This continuous implantable approach fundamentally differs from consumer wearables by prioritizing uninterrupted rhythm surveillance over broad accessibility.

A distinct outcome-oriented paradigm is seen in the STROKESTOP II trial, which integrated biomarker-based risk enrichment (NT-proBNP) with single-lead ECG screening. While new AF was identified in 2.4% of screened individuals, this strategy did not yield a statistically significant reduction in stroke or systemic embolism at the population level, in contrast to detection-only wearable studies that primarily report diagnostic performance. Nevertheless, the favorable outcomes observed in individuals with low NT-proBNP illustrate that screening impact is highly dependent on baseline cardiovascular risk rather than detection capability alone [20].

At the algorithmic ECG level, the AI Watch study illustrates how deep neural network analysis applied to Apple Watch single-lead ECG can outperform native smartwatch algorithms by maintaining high sensitivity (91%) and specificity (95%) while dramatically reducing inconclusive readings [10].This positions AI-enhanced ECG as an intermediate solution that narrows the gap between consumer-grade convenience and near–clinical-grade diagnostic reliability.

Overall, PPG-based wearables maximize population reach with high technical performance but depend heavily on secondary confirmation and threshold definitions; single-lead ECG devices enhanced with AI improve diagnostic certainty at lower scale; implantable monitors offer continuous, high-fidelity surveillance with limited applicability to mass screening; and biomarker-guided ECG strategies highlight that detection alone is insufficient to guarantee downstream clinical benefit.

2. Algorithmic Innovations for Resource-Constrained and Edge/Wearable Deployment

Recent advances in wearable and edge intelligence have fundamentally redefined how electrocardiographic analysis is implemented under strict constraints of computation, memory, energy consumption, and real-time responsiveness. Within this context, Table2 combines a series of representative deep learning strategies that explicitly target arrhythmia detection and ECG interpretation in resource-limited environments. The studies summarized in this table span a broad spectrum of design philosophies, ranging from signal reconstruction and representation-level compression to TinyML-based architectural optimization, interpretable lightweight CNNs, and federated adaptive intelligence. Together, they illustrate how algorithmic innovation is progressively enabling continuous, autonomous, and clinically meaningful ECG analytics directly at the wearable or edge level.

The earlier studies in Table2 approach resource limitations primarily by modifying how ECG information is represented rather than by aggressively shrinking the network itself. Kwon (2022) increases diagnostic depth by reconstructing full 12-lead ECG signals from only two smartwatch leads using a GAN–CNN pipeline [11]. This allows richer clinical interpretation without adding new sensors, but the system is still dependent on hospital-based computation and has not yet demonstrated true autonomy in everyday wearable use [11]. Vu (2023) follows a different path by converting ECG signals into 2D image-like inputs and processing them with a compact CNN [18].

This design enables real-time inference on wearable edge hardware, yet the lack of reported sensitivity and specificity makes it difficult to judge its clinical reliability alongside physiology-based models [18]. Later works move more decisively toward direct architectural compression and energy-aware deployment. Hizem (2025) reduces the full arrhythmia detection pipeline to an ultra-compact TinyML model of roughly 193 KB through pruning and quantization, allowing continuous operation on low-power IoMT platforms [22]. This level of structural minimization contrasts with Zihao Li (2025), where compression is achieved through knowledge distillation and automated multi-lead fusion [23]. That model reaches extremely high QRS detection performance with only a few thousand parameters, but its focus remains limited to waveform localization rather than full rhythm interpretation [23].

At a higher level of clinical performance under constrained computation, Kraft (2025) and Monachino (2025) represent two complementary optimization philosophies. Kraft employs a lightweight 1D ConvNeXtV2 architecture that achieves very high atrial fibrillation detection sensitivity while also incorporating interpretability into the model design, which supports clinical trust under limited computational budgets [8]. Monachino, by contrast, focuses on overcoming data scarcity rather than minimizing parameters alone. By applying transfer learning to compact residual CNNs, this approach achieves exceptionally high specificity for life-threatening ventricular arrhythmias in wearable OHCA vests, even with a small fine-tuning dataset [24]. One prioritizes transparent inference under hardware constraints, while the other prioritizes diagnostic reliability under limited data availability.

A different direction is introduced by Alghieth (2025), where lightweight inference is combined with adaptive and federated learning [25] so ethical approval was not required. The hybrid Transformer–CNN architecture enables collaborative learning across distributed wearable nodes and supports continuous system-level adaptation [25] so ethical approval was not required. This flexibility, however, comes with increased memory usage and higher battery consumption, placing this model closer to the practical upper limit of what can still be sustained in long-term edge deployment [25]so ethical approval was not required.

3. Foundation-Scale and Multimodal Large-Language/Pre-Trained Models (Relevant to Future Wearable Use)

Theme 3 will focus on foundation-scale and multimodal pre-trained models with direct relevance to future wearable cardiovascular monitoring. These emerging approaches move beyond conventional task-specific classifiers by leveraging large heterogeneous datasets to enable multi-disease inference, cross-modality prediction, and scalable deployment on resource-constrained single-lead wearable devices [21, 26, 27]. The studies presented in Table3 are positioned at the intersection of large-scale representation learning and portable clinical monitoring, highlighting how pre-training strategies reshape the diagnostic capacity of wearable ECG systems across arrhythmia detection, structural heart disease inference, and long-term risk prediction [21, 27]. The studies presented in Table3 reflect a clear expansion in both the clinical ambition and technical capacity of wearable-compatible cardiovascular AI, while differing in how broadly each model defines its diagnostic role [21, 26, 27].

 Monachino (2025) follows a focused strategy centered on long-term arrhythmia detection, where transfer learning from a moderately large dataset enables high diagnostic performance despite very limited task-specific training data. The model is optimized for real-time wearable use, emphasizing fine temporal resolution and exceptionally high specificity to support reliable alert generation in continuous monitoring settings [24]. In contrast, Jun Li’s ECGFounder is designed as a general-purpose foundation model trained on more than ten million ECG recordings. Rather than targeting a single clinical endpoint, it simultaneously supports over 150 diagnostic tasks across both 12-lead and single-lead ECG formats. This breadth shifts the role of wearable ECG devices from specialized arrhythmia detectors toward adaptive platforms capable of integrated rhythm analysis, demographic inference, and prognostic evaluation within a single unified framework [21]. Knight (2025) further broadens the functional scope of wearables by introducing cross-modality learning through the Wearable-Echo-FM model. By transferring knowledge from echocardiography to single-lead ECG, this approach enables inference of structural cardiac phenotypes such as ejection fraction and valvular disease directly from wearable signals. This capability places wearable devices closer to imaging-informed decision support, rather than limiting their role to front-line screening [26].

 Aminorroaya (2025) adopts a different but complementary direction by focusing on prospective disease risk rather than concurrent diagnosis. ADAPT-HEART uses large-scale real-world ECG data to estimate future structural heart disease development and demonstrates stable performance in community-based cohorts. This predictive orientation extends wearable applications into preventive cardiology and population-level risk management, distinguishing it from both multimodal imaging inference and broad diagnostic generalization [27].

 Across all four models, strong discriminative performance is consistently reported, yet each system reflects a different balance between diagnostic precision, task breadth, modality integration, and temporal prediction horizon. What unites them is the reliance on large-scale pre-training as the key mechanism that enables clinically meaningful inference from limited, noisy single-lead wearable ECG signals.

4. Small-to-Medium Real-World Deployments, Prototype Validations & Predictive Models

Theme 4 summarizes recent small-to-medium–scale real-world deployments, prototype validations, and predictive modeling studies focused on wearable and portable cardiovascular monitoring technologies. These studies examine how emerging sensing systems perform beyond controlled laboratory conditions, with attention to diagnostic accuracy, continuous monitoring feasibility, early risk prediction, and signal reliability in real-world environments. Rather than concentrating solely on large-scale population screening, the works presented in this table capture diverse clinical intentions, ranging from disease detection and waveform-level signal analysis to multimodal system integration and consumer-grade device validation. The following Table4, represents the theme 4 findings [10, 13, 14, 28, 29, 30].

The studies presented in Table4 reflect the broad and evolving role of wearable technologies in cardiovascular monitoring, where electrocardiography and photoplethysmography form the core sensing modalities supporting different clinical goals. ECG-based systems are primarily used for disease detection and structural or functional cardiac assessment, while PPG-based approaches increasingly support continuous and predictive monitoring in daily-life settings [10, 28, 29].

 Among the ECG-focused investigations, Kwon (2022) and Aminorroaya (2025) apply wearable ECG systems to heart failure and structural heart disease screening across very different deployment scales [11, 27]. Kwon reports high diagnostic performance for heart failure with reduced ejection fraction in a hospital-based smartwatch cohort, achieving an AUROC of 0.934. Aminorroaya extends this approach to a much larger population across community hospitals and external cohorts, involving more than 99,000 participants and maintaining strong predictive performance with an AUROC of 0.879 [27].

 Despite differences in scale, both studies share an important limitation, as wearable ECG outputs still require echocardiographic confirmation, which confines these systems mainly to screening and risk stratification rather than fully independent diagnosis [11, 27]. A different direction is taken by Gavidia (2024), who shifts the focus from disease detection to early prediction. Using R–R intervals derived from smartwatch-based PPG signals, this study predicts atrial fibrillation onset around 30 minutes in advance. This predictive framing sets the work apart from ECG-based classification studies and highlights the growing potential of low-burden consumer devices for preventive cardiovascular care rather than post-event detection [28].

 At a more fundamental signal-processing level, Zihao Li (2025) addresses waveform-level QRS detection using multi-lead ECG data from standard arrhythmia databases. With F1 scores above 99% on both the MIT-BIH and INCART datasets, together with a very compact model architecture, this study demonstrates strong algorithmic efficiency and cross-database robustness. However, unlike the other works in the table, its contribution is predominantly technical, supporting upstream signal extraction rather than direct clinical decision-making [23].

 Moving toward integrated sensing systems, Chen (2025) explores wearable bioelectronic platforms that combine ECG, PPG, pulse signals, seismocardiography, and bioimpedance in small cohorts of coronary heart disease and heart failure patients. The results show that combining multiple signal modalities consistently improves diagnostic accuracy compared with single-sensor approaches. At the same time, the study brings attention to continuing challenges related to system scalability, data privacy, and regulatory approval, which currently limit wider clinical deployment [31]. The performance of consumer-grade devices in real clinical settings is evaluated by Vermunicht (2025) in cardiac rehabilitation patients using both ECG- and PPG-based wearables. After applying artifact removal procedures, PPG-derived heart rate measurements show moderate correlation and improved accuracy. However, the small sample size and the exclusive emphasis on heart rate, rather than rhythm or arrhythmia detection, reduce the broader clinical applicability of the findings [29].

 Overall, a clear progression from controlled database-based signal analysis and early prototypes toward real-world screening, predictive monitoring, multimodal integration, and consumer-device deployment. ECG continues to serve as the backbone for clinically oriented disease detection, while PPG is emerging as a practical solution for continuous and anticipatory monitoring beyond traditional medical environments [13, 28, 29]. Across all studies, shared challenges related to validation against gold standards, real-world signal robustness, scalability, and regulatory integration continue to shape the translational path of wearable cardiovascular technologies [13, 14, 31].

**Table:** Table1. Lightweight AI models for on-device arrhythmia detection in wearables

First Author	Year	Study name / Device	Population (N)	Signal	Primary endpoint / AF detection method	Sensitivity / PPV for AF	Specificity / NPV	Key real-world finding / Limitation
Perez MV	2019	Apple Heart Study / Apple Watch	419,297	PPG + tachogram AI	Irregular pulse notification → ECG patch confirmation	98.3% (sensitivity vs. patch ECG) / PPV 84% (for confirmed AF)	99.6%	First FDA clearance for consumer AF screening; highlights low confirmation rate (0.5%) and high false positives in ambulatory use.
Dörr	2019	WATCH AF Trial / Samsung smartwatch + AliveCor iECG	672 enrolled, 508 analyzed	PPG + iECG	Accuracy of PPG for AF detection vs. iECG / PPG-based algorithm + cardiologist-interpreted iECG confirmation	93.7% / 97.8%	98.2% / -	Earliest prospective validation of consumer smartwatch PPG; excellent metrics despite motion / High uninterpretable rate 21.8% due to poor contact; elderly hospitalized cohort
Guo Y	2021	Huawei Heart Study / Huawei devices	187,966	PPG	AF detection / PPG + ML	Not reported / PPV 91.6%	Not reported	Feasible screening; high PPV / General population focus
Lubitz SA	2022	Fitbit Heart Study / Fitbit wearables	455,699	PPG	PPV of IHRD for concurrent AF / IHRD (≥11 irregular 5-min tachograms; ≥30 min irregular) → ECG patch (7 days)	98.0% / 98.0%	99.7% / -	High PPV for concurrent AF; identifies undiagnosed AF / IHRD only for ≥30 min episodes; healthy bias (median age 47, 71% female)
Olson JA	2023	LUX-Dx PERFORM / LUX-Dx ICM	369 (interim)	Subcutaneous ECG	ICM remote monitoring and programming / Arrhythmia detection	Not reported	Not reported	Infection rate 0.8%; 94% daily transmission; remote programming feasible / Interim results only
Gudmundsdottir KK	2024	STROKESTOP II / Zenicor ECG	28,712 (participation 6,843)	Single-lead ECG	Stroke/systemic embolism reduction / NT-proBNP then ECG screening	Not reported / 2.4% new AF detection	Not reported	No reduction in stroke/SE (HR 0.96); low NT-proBNP safe (HR 0.59) / Moderate participation (49%)
Fiorina L	2024	AI Watch studies / Apple Watch	400(cardiac patients)	Single-lead ECG	AF detection / DNN on SW ECG vs 12-lead	91% (95% CI 85-95) / Not reported	95% (95% CI 91-97) / Not reported	Low inconclusive (1% vs 22%); better than Apple app / Monocentric; mostly male

**Table:** Table2. Lightweight algorithmic innovations designed for on-device or edge deployment in wearables

First Author	Year	Model Type / Architecture	Target Platform / Device	Parameters / Power	Signal	Target Arrhythmias	Sensitivity	Specificity / Accuracy	Main Innovation / Key Limitation
Kwon	2022	GAN (ECGT2T) + CNN (residual blocks)	Smartwatches (e.g., Apple/Galaxy)	Not specified	ECG (2-lead to 12-lead)	HFrEF (rhythm-related)	0.897 (overall); 0.974 (Watch A)	Spec: 0.860 / AUC: 0.934	Inn: 12-lead synthesis from wearables. Lim: Hospital-only validation; no home use.
Vu	2023	2D CNN (3 conv layers, inspired by AlexNet/VGG)	Custom wearable sensor; edge devices	Filters: 8-32; Not specified	ECG (2D images)	General arrhythmias (9 classes)	Not specified	Acc: ~90%; Inference: 20 ms	Inn: Real-time 2D image ECG on wearables. Lim: No sens/spec; healthy-only real test.
Gavidia	2024	Deep Learning (RNN; RRI-to-image)	Smartwatches (RRI-accessible)	Not specified	ECG (R-to-R intervals)	AF (prediction)	Not specified	Acc: 83% / F1: 85%	Inn: 30.8 min AF warning. Lim: Retrospective; no prospective validation.
Hizem	2025	CNN (conv, pooling, dropout; pruned/quantized)	Raspberry Pi/Arduino (IoMT)	~193 KB (opt)	ECG	Arrhythmias (AF, PVCs, etc.)	92.89% (opt)	Acc: 92.89% (opt) / F1: 93.96%	Inn: TinyML edge anomaly detection. Lim: Single dataset; approx power.
Zihao Li	2025	LDM (distillation) + U-Net (down/up-sampling)	Wearable ECG devices	5216 params	ECG (multi-lead)	QRS (for arrhythmias)	Se: 99.92% (MITBIH)	Acc: Not spec / F1: 99.83%	Inn: Auto multi-lead fusion; scaling op. Lim: QRS-only; manual scaling factor.
Monachino	2025	CNN (ECGnet residual; transfer learning)	Wearable systems (OHCA vest)	Reduced versions (M/S)	ECG (single-lead)	LTAs (VF, VT)	92.68%	Spec: 99.48% / Not spec	Inn: Transfer learning for scarce data; confidence alerts. Lim: Small fine-tune set; transitory phases weak.
Kraft	2025	1D ConvNeXtV2 CNN (4 stages, GRN)	Wearables/bedside monitors	770k params; 46 MFLOPs	ECG (single-lead)	AFib/AFL	0.982 (MIT-AFDB)	Acc: Not spec / F1: 0.986	Inn: Interpretable lightweight CNN. Lim: Higher complexity vs baselines; no pretraining.
Alghieth	2025	Hybrid Transformer-CNN (MHSA, denoising)	Raspberry Pi; wearables/edge	30 MB mem; ~15% battery/12h	ECG (single-lead)	Anomalies (AF, VT, etc.)	96.8%	Acc: 98.2% / F1: 97.5%	Inn: Adaptive detection; federated learning. Lim: Noise reduces perf; binary-only.

**Table:** Table3. Foundation-scale and multimodal pre-trained models with direct relevance to future wearable arrhythmia detection

First Author	Year	Model name	Training data	Signal(s)	Tasks	Key performance	Relevance to wearables
Monachino	2025	Transfer learning-based deep learning model	72,952 recordings (pre-training) + 102 recordings (fine-tuning)	Single-lead ECG	LTA detection	Sensitivity 92.68% / Specificity 99.48%	Transfer learning from massive dataset to small LTA data; mitigates data scarcity for wearable LTA detection; granularity of 1.28 seconds for real-time alerts.
Knight	2025	Wearable-Echo-FM	Large ECG-echo pairs (millions of recordings)	Single-lead ECG + echo pairs	Predict structural heart diseases (EF, valve disease) from ECG	AUC 0.89–0.94	Foundation model for single-lead wearable ECGs; scales to portable devices; enables cross-modality prediction (ECG to echo findings) via knowledge distillation.
Jun Li	2025	ECG Foundation Model (ECGFounder)	Over 10 million ECGs	12-lead + single-lead ECG	150+ diagnostic tasks (arrhythmias, demographics, events)	AUROC >0.95 for 80 diagnoses	General-purpose pre-trained model; extends to arbitrary single-lead ECGs for wearables; fine-tunable for mobile monitoring.
Aminorroaya	2025	ADAPT-HEART	266,740 ECGs from 99,205 patients	Single-lead ECG	Detect/predict SHDs (e.g., low EF, valvular disease)	AUROC 0.879 (95% CI 0.870–0.888)	Noise-resilient for portable/wearable devices; predicts future SHD risk (2.8-5.7x higher with high probability); validated in community cohorts like ELSA-Brasil.

**Table:** Table4. summarizes these studies, focusing on deployment, outcomes, and constraints.

First Author	Year	Device / Setting	Population	Signal	Primary outcome	Key result	Limitation
Kwon	2022	Smartwatch / Hospital B	755 patients	2-lead ECG	Detect HFrEF (EF <40%)	AUROC 0.934 (95% CI 0.913–0.955)	Requires echocardiography for validation; hospital-based, not fully community; sensitivity 0.897, specificity 0.860.
Gavidia	2024	Smartwatches / Test data	Test data cohorts	R-R interval from PPG	Predict AF onset	Predicts AF 30.8 min in advance; 83% accuracy, 85% F1 score	Has the potential to lower interventions and costs by early AF detection; uses R-to-R interval signals for monitoring, accessible via smartwatches.
Aminorroaya	2025	Wearable/portable devices / Community hospitals + ELSA-Brasil	99,205 patients (development); external cohorts	Noisy single-lead ECG	Detect/predict SHDs	AUROC 0.879 (95% CI 0.870–0.888)	Limited role in community-based screening; high vs. low probability conferred a 2.8- to 5.7-fold increase in the risk of future SHD.
Chen	2025	Wearable bioelectronics / Digital healthcare prototypes	Small cohorts (e.g., CHD/HF patients)	ECG + PPG + pulse + SCG + bioimpedance	Continuous monitoring / diagnostics	Fusion improves accuracy vs single modality	Challenges in scalability, privacy, and regulatory compliance; the integration of artificial intelligence (AI) with wearable bioelectronics is revolutionizing digital healthcare.
Zihao Li	2025	Wearable ECG devices / Databases (MITBIHA/INCART)	MITBIHA/INCART cohorts	Multi-lead ECG	QRS detection	F1 score 99.83 on MITBIHA	Parameter count of only 5216; our method provides a novel idea for universal multi-lead QRS detection; in the cross-database pattern, our approach maintains a strong performance with an F1 score of 99.22 on the INCART database.
Vermunicht	2025	Multiple consumer devices (Polar H10, Fitbit Inspire 2) / Cardiac patients in CR	15 patients with AF/HF/CAD	HR from ECG/PPG	Continuous HR accuracy + artefact removal	PPG improved after ARP: correlation 0.75	Small cohort (N=15); the procedure removed nearly one-third of unreliable data, achieving an 81% accuracy; focus on HR, not rhythm.

## Discussion

Across the evaluated evidence, four interrelated dimensions define the current landscape of AI-enabled wearable ECG and PPG systems for arrhythmia detection: technical accuracy, computational feasibility, real-world implementation, and clinical impact [15, 17, 32]. Together, these dimensions illustrate both the maturity of the technology at the algorithmic level and the constraints that continue to shape its clinical translation [33, 34].

Under controlled conditions, diagnostic performance consistently reaches high levels, with sensitivities and specificities frequently exceeding 90% in curated datasets and technical validation frameworks [6, 9]. In contrast, performance in ambulatory environments is systematically affected by motion-related artifacts, variable electrode–skin contact, signal noise, and inter-individual physiological differences [13, 29]. These factors contribute to reduced specificity and higher inconclusive recording rates, indicating that laboratory-level accuracy does not directly translate into uniform real-world reliability [35, 36].

Parallel to these limitations, substantial progress has been achieved in algorithmic design for resource-constrained environments [22, 25]. Lightweight convolutional architectures, hybrid transformer–CNN models, quantization and pruning strategies, and federated learning frameworks have collectively enabled near–real-time inference with moderate power consumption [18, 23]. These developments establish a practical foundation for continuous on-device monitoring [8, 24]. However, many proposed systems remain evaluated primarily in retrospective datasets or narrowly defined cohorts, which restricts confident extrapolation to broader clinical populations [32, 33].

Large prospective population-based trials confirm the feasibility of atrial fibrillation screening using consumer-grade wearables, with consistently high positive predictive values and acceptable long-term adherence [2, 4, 5]. These studies demonstrate that large-scale passive detection in unselected populations is operationally achievable [3, 7]. At the same time, outcome-oriented investigations have not demonstrated consistent population-level reductions in stroke, systemic embolism, or mortality [12, 20]. This discrepancy highlights the distinction between detection capability and clinical effectiveness, emphasizing that screening benefit is strongly modulated by baseline risk, downstream care pathways, and treatment adherence rather than by algorithmic performance alone [1, 14].

The emergence of foundation-scale pre-trained models and multimodal learning systems further broadens the functional scope of wearable monitoring [21, 26, 27]. By enabling structural heart disease inference, longitudinal risk stratification, and cross-modality prediction from single-lead ECG signals, these approaches reposition wearables from single-task detectors toward multipurpose cardiovascular assessment platforms [26, 37]. Nevertheless, their clinical applicability under real-world constraints of power consumption, regulatory oversight, interpretability, and workflow integration has not yet been fully established [31, 38].

## Conclusion

Several prerequisites must therefore be addressed before routine clinical deployment can be justified at scale. These include sustained signal robustness in ambulatory conditions, transparent and reproducible algorithmic pipelines, systematic validation across diverse demographic and comorbidity profiles, regulatory standardization, and large multicenter trials focused on hard clinical outcomes rather than detection metrics alone [1, 17, 38]. In summary, wearable ECG- and PPG-based systems supported by advanced computational methods now demonstrate high diagnostic capability for atrial fibrillation and related rhythm disturbances under controlled and selectively validated real-world conditions [10, 15]. Population-scale screening is feasible and diagnostically effective for identifying previously unrecognized disease [2, 4]. However, limitations in real-world signal stability, generalizability, and outcome-level benefit currently constrain broader clinical adoption [13, 20]. Resolution of these challenges will determine whether wearable cardiovascular technologies evolve from effective screening instruments into integrated components of preventive cardiology and routine clinical care [1, 17].

### Limitations

Several methodological and evidentiary limitations should be acknowledged when interpreting the findings of this review. The most important limitation arises from the substantial heterogeneity across the included studies, encompassing device types (PPG-based wearables, single-lead ECG smartwatches, implantable cardiac monitors), signal modalities, patient populations, validation standards, and reported outcomes. This variability precluded formal quantitative meta-analysis and limits direct numerical comparison of diagnostic performance across studies. Although several large-scale prospective population trials were included, a considerable proportion of the evidence base, particularly within recent algorithmic and multimodal developments, relies on retrospective analyses, controlled signal databases, or small- to medium-sized cohorts. Models demonstrating very high performance on curated datasets such as MIT-BIH and INCART may not fully reflect performance under real-world ambulatory conditions characterized by motion artifacts, environmental noise, and variable skin contact. Demographic representation also remains an important constraint. Many datasets show imbalances with respect to age, sex, ethnicity, and comorbidity profiles, potentially leading to optimistic estimates of diagnostic performance and limited generalizability to older, multi-morbid, or underserved populations. Even large community-based trials may be affected by participation bias favoring healthier and more technology-literate individuals. Reproducibility is further limited by restricted transparency in proprietary commercial algorithms. In several large consumer platforms, key aspects of signal preprocessing, artifact suppression, and irregular rhythm detection thresholds remain undisclosed, hindering independent validation and standardized benchmarking across vendors. Finally, while foundation-scale and multimodal models extend wearable applications beyond arrhythmia detection toward structural heart disease prediction and long-term risk stratification, clinical validation of these systems remains limited, and their feasibility under the computational, power, and regulatory constraints of wearable deployment is not yet fully established. In addition, unpublished industry data, negative studies, and non-English publications may not be fully captured despite the broad search strategy.

## Conflict of Interest

The authors declare no conflict of interest.

## AI Disclosure Statement

During the preparation of this manuscript, the authors used ChatGPT, OpenAI company for language editing, grammar improvement, and liboberry.com for reference management. After its use, the authors thoroughly reviewed, verified, and revised all AI-assisted content to ensure accuracy and originality. The authors take full responsibility for the integrity and final content of the published article.
